# Systemic Immunotherapy for the Treatment of Brain Metastases

**DOI:** 10.3389/fonc.2016.00049

**Published:** 2016-03-09

**Authors:** Justine V. Cohen, Harriet M. Kluger

**Affiliations:** ^1^Section of Medical Oncology, Department of Medicine, Yale Cancer Center, New Haven, CT, USA

**Keywords:** immunotherapy, brain metastases, melanoma

## Background

Significant progress has been made in the treatment of selected malignancies with immune-modulating antibodies. Phase III trials of anti-CTLA-4 in melanoma and anti-PD-1 in melanoma, renal cell carcinoma (RCC), and non-small cell lung cancer (NSCLC) showed improved overall survival (OS) compared to standard therapies (1–5). As a result, immune checkpoint inhibitors are now approved for the treatment of these diseases. Blockade of CTLA-4 (ipilumimab and tremelimumab), PD-1 (nivolumab, pembrolizumab, pidilizumab and others), and PD-L1 [BMS 936559 (6), durvalimumab (7), and atezolizumabes (8–11)] can produce durable responses in patients with metastatic cancer. Clinical trials with these agents, alone and in combination, are ongoing. Moreover, additional immune checkpoint modulators are in pre-clinical and clinical development. Other approved immunotherapies include high-dose bolus interleukin-2 (IL-2), interferon alpha-2b, and Sipuleucel-T. There are limited data, however, on the impact of immunotherapy in patients with measurable metastatic disease to the brain. Registration trials of immune therapies excluded patients with active brain metastases based on a historical poor prognosis in this patient population coupled with uncertainty about the ability of the drugs to cross the blood brain barrier (BBB). These active therapies might however have benefited patients with microscopic brain deposits.

Brain metastases were historically managed with whole brain radiation therapy (WBRT) or surgical resection, depending on the size, number, histology, symptoms, and location. The availability of high-resolution magnetic resonance imaging (MRI) and stereotactic radiosurgery (SRS) to small, emerging lesions has improved local lesional control. These modalities allow higher doses of radiation. In many institutions, WBRT is reserved for patients with multiple or larger lesions not amenable to SRS (12, 13). These treatments are not without limitations and consequences. For example, WBRT has been associated with cognitive decline, while SRS can result in radiation necrosis, cerebral edema, and delayed tumor hemorrhage (14, 15). More often, however, focal therapies are limited in efficacy due to distant cerebral relapse and lack of treatment of microscopic tumor foci not evident on imaging. As new systemic treatments, particularly immune-modulating agents, show prolonged survival of patients with aggressive extra-cerebral disease, these drugs need to be assessed for efficacy in active brain metastases. There are a number of ongoing investigations to determine if these antibodies cross the leaky BBB found in tumors despite their size (16, 17). Alternatively, although brain metastases might contain pre-existing tumor infiltrating lymphocytes (TILs), immune modulation induced by these agents may allow cytotoxic T cells into the tumor microenvironment in the brain, resulting in antitumor immunity. Several lines of evidence suggest that T cells within the tumor microenvironment are responsible for the responses seen with these therapies (18, 19). To date, there have been no published pharmacokinetic or pharmacodynamic studies in on-treatment brain tissue to allow determination of drug penetration into the tumor, primarily due to the difficulty accruing patients to trials requiring brain biopsies, particularly from patients who are responding to therapy. Although animal studies have been done, drug distribution and T cell activation might not reflect that of humans.

Metastatic melanoma is the solid tumor with the highest propensity for dissemination to the brain (20). The only chemotherapy widely used for melanoma known to definitively cross BBB is temozolamide, which induced responses in 7% of melanoma brain metastasis patients (21). Other anti-neoplastic drugs that cross the BBB include fotemustine, etoposide, cisplatin, vinblastine, and motoxantrone and can be used depending on tumor cell sensitivity (22–26). Targeted therapies such as erlotinib, afatinib, and lapatinib have also shown evidence of ability to cross the BBB (27–29).

## Preclinical Data

The ability of immune-modulating antibodies to cross the BBB and control brain metastases is the subject of ongoing investigations. In primary CNS tumors, preclinical data with immune-modulating antibodies have shown promise. In mice with SMA-650 intracranial tumors, anti-CTLA-4 was tolerated well (30). An increase in CD4+ cells and decrease in T regs prolonged survival in these animals. Similarly, PD-1 blockade combined with radiation was tested in mice with GL261 intracranial tumors and showed improved survival (31). The combination of PD-1 and CTLA-4 inhibitors similarly showed improved survival in animal models (32). These examples suggest that BBB drug penetration in tumors might be obtainable, for primary CNS tumors and for metastatic tumors, although this remains to be verified in humans with each drug and tumor type.

## Clinical Data

High-dose IL-2 was one of the first immune-modulating agents to demonstrate activity in melanoma and RCC. There have not been any formal trials of IL-2 specifically for patients with brain metastases. A retrospective series reported a response rate in active brain metastases lower than expected for extra-cerebral disease, however without excessive toxicities (33). One of the first studies to investigate the effect of immunotherapy on brain metastases in patients with metastatic melanoma was a retrospective analysis of the phase II trial with ipilimumab, which reported 5 of 12 patients were responders (34, 35). Following this observation, a phase II trial of ipilimumab specifically for patients with brain metastases from melanoma opened (36). Results of 72 patients accrued showed prolonged OS, particularly notable in asymptomatic patients. These findings were confirmed in an expanded access protocol of ipilimumab with a 20% 1-year OS in patients with stable, asymptomatic brain metastases (37). Based on these promising results, the Italian Network for Tumor Biotherapy (NIBIT) designed a phase II trial of ipilimumab in combination with fotemustine (NIBIT-M1) with twenty asymptomatic patients with brain metastases. Stable disease or partial response was seen in 25% and another 25% had complete response in the brain (38, 39).

A follow-up randomized trial (NIBIT-M2) was subsequently initiated for patients with untreated melanoma brain metastases comparing fotemustine monotherapy, fotemustine plus ipilimumab 10 mg/kg and ipilimumab 3 mg/kg + nivolumab 10 mg/kg (NCT02460068). Objectives include OS, safety, disease control rate (intra and extra-cerebral) objective response rate, duration of response, and progression-free survival. This study will also examine quality of life. Various groups are studying the effect of immune-modulating agents alone and in combination with other therapies for the treatment of brain metastases from melanoma. For example, ipilimumab and nivolumab or nivolumab monotherapy is being studied in a large multi-arm phase II trial (NCT02320058 and NCT02374242) and combinations of ipilimumab with various forms and schedules of radiation are being investigated (NCT01703507, NCT01950195 and NCT02097732). Results of these trials are pending.

A phase II trial of pembrolizumab for patients with metastatic melanoma or NSCLC and untreated brain metastases is ongoing. Preliminary results from this trial were presented at ASCO 2015 (NCT02085070) (40, 41). In this two-arm study, patients are eligible if they have at least 1 untreated or progressive brain metastasis (5–20 mm), not requiring steroids and are without neurological symptoms. Patients in the melanoma arm require brain metastasis biopsy or resection of metastatic brain lesion prior to starting therapy or availability of previously resected brain lesions for correlative studies. Patients in the NSCLC arm are required to have PD-L1 positive tumors. In the NSCLC arm, 11 patients were evaluable for response as of June 2015. Brain metastasis response rate was 45%, and systemic response rate was 45%. Only one patient with a systemic response had disease progression in the brain, and two patients with disease progression as their best systemic response were unevaluable in the brain due to rapid systemic progression. The duration of response in the brain was at least 12 weeks for four of five responders, and all responses were ongoing at the time of data analysis (40). In the melanoma arm, 18 patients were accrued at the time of analysis. Four patients were unevaluable due to rapid extra-cerebral progression or hemorrhage, and one was too early for response evaluation. Four patients achieved partial response, three had stable disease, and seven had disease progression (two with mixed response and one with histologically demonstrated pseudoprogression). Response in the body was largely concordant with brain response, although in some cases brain response occurred after extracerebral response. Response in the brain was ongoing at 4+, 6+, 6+, and 11+ months (41).

Studies completed to date suggest that immune checkpoint inhibitors have activity in the brain that might be similar to that of extra-cerebral sites (42). In the phase II study of ipilimumab brain metastases activity in asymptomatic patients was similar to that of patients without brain metastases with a disease control rate of 24 and 27%, respectively. The 1- and 2-year progression-free survival were 31 and 26%, respectively (36, 43). The NIBIT-M1 study described above confirmed these findings with an immune-related disease control rate for patients with brain metastases of 50% compared with 46.5% of the entire treated population. Interim data from our phase II trial of pembrolizumab in patients with metastatic melanoma and NSCLC with untreated brain metastases showed that all responses in the melanoma arm were concordant, while three or four in the NSCLC arm were concordant (40, 41). Results suggest that immune-modulating agents may have similar durable responses in the brain as seen systemically, and support use of systemic therapy alone or in combination with focal therapy (SRS or surgery) in the treatment of brain metastases from immune therapy responsive diseases such as melanoma and lung cancer.

There are data to suggest that responses might be further improved by combining immune checkpoint inhibitors with radiation. Several studies have evaluated the combination in other disease sites (44–47). A number of mechanisms have been described explaining the combined effect; radiation upregulates inflammatory cytokines (i.e., TNFα, IFN-γ, and CXCL16), promoting tumor detection and facilitating T cell infiltration (48, 49). Radiation can upregulate PD-L1 (50). The abscopal effect, in which local radiation is thought to cause a systemic response resulting in shrinkage at distant sites, further supports the use of radiation combined with immune-modulating agents (51). Knisely et al. published a series of patients with metastatic melanoma with brain metastases who achieved a median survival of 21.3 months if they received ipilimumab and SRS versus 4.9 months if they underwent SRS but did not receive ipilimumab (44). Mathew et al. looked at a similar population with 25 patients receiving both ipilimumab and SRS versus 33 patients receiving SRS alone (46). The analysis did not show a significant benefit in 6-month OS between the two groups, although this was not a randomized trial and the groups were not balanced. Lastly, Silk et al. reported improved OS in patients receiving ipilimumab and SRS (47). Exploratory analysis within the same study showed no increase in OS with the addition of ipilimumab to WBRT. The timing of administration of concurrent immune checkpoint inhibitors and radiation has not yet been determined. Kiess et al. found increased rates of progression if patients were treated with SRS before or during ipilimumab compared with those who received SRS after systemic therapy (52). Future studies will provide insight into the optimal timing for combining radiation and immune-modulating therapies, such as NCT02097732, which is investigating SRS to brain metastases before or in the middle of ipilimumab induction.

**Figure 1:**
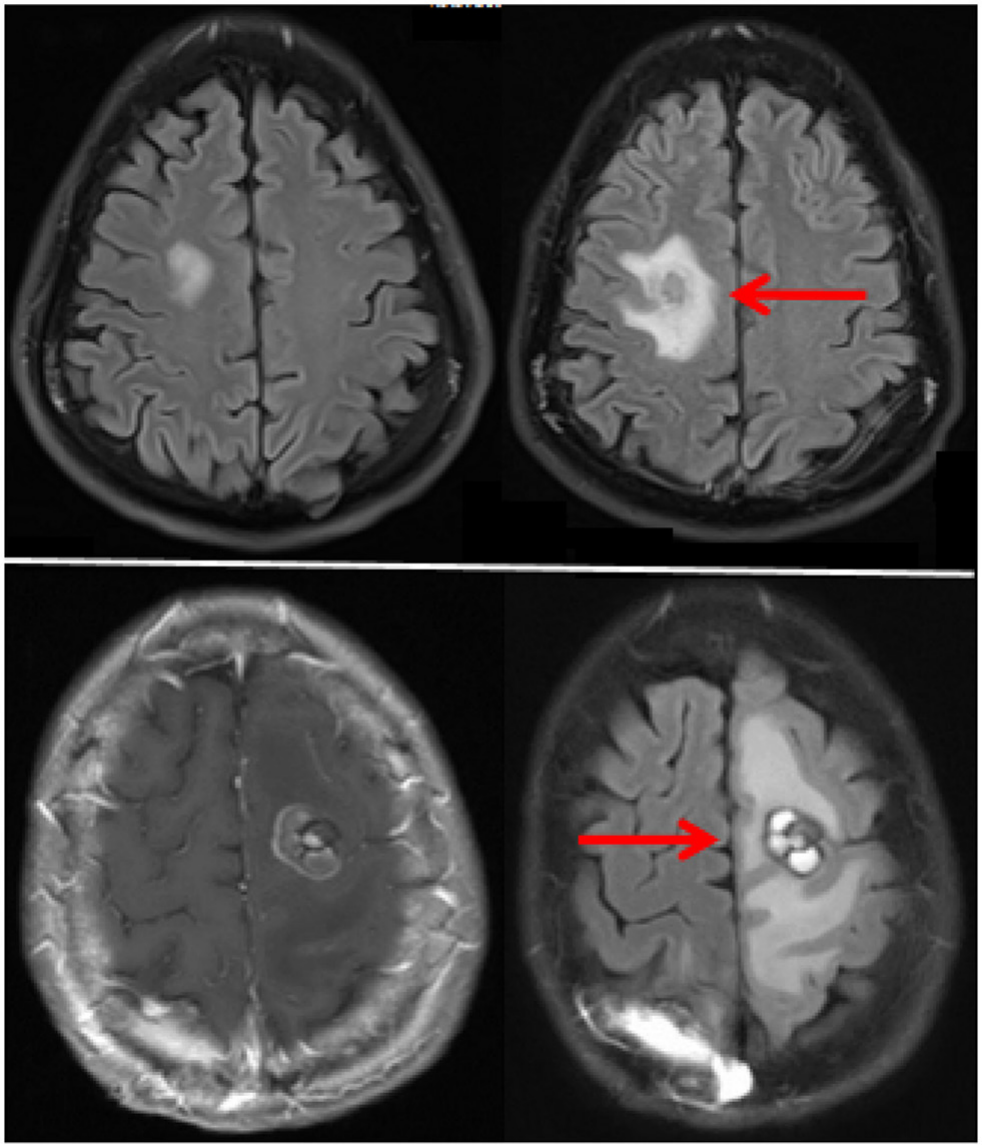
**MRI FLAIR images of two patients with perilesional while receiving pembrolizumab**. The top and bottom frames represent the two separate patients. Images prior to therapy are on the left and after therapy on the right.

Toxicities unique to central nervous system metastases, such as vasogenic edema and tumor necrosis represent an additional challenge. Early recognition of potential symptoms is essential. One of the challenges in treating brain metastasis patients with immune therapy is management of neurological symptoms, which might be from perilesional edema, intralesional hemorrhage, necrosis most commonly seen in previously irradiated lesions, or tumor growth due to treatment failure. Examples of perilesional edema seen on FLAIR images before and on therapy in two patients receiving pembrolizumab are shown in Figure 1. Both patients responded well to transient steroids and remain on pembrolizumab with good disease control for over a year. Depending on the size and location of the brain metastasis, patients might require surgical intervention due to neurologic symptoms. Moreover, it is sometimes impossible to determine whether lesions enlarge on study due to inflammation, necrosis, or tumor growth, and current imaging modalities can be inadequate (53). Our institutional experience suggests that despite the indisputable benefit of systemic immune therapy in some tumor types, radiation necrosis occurs with greater frequency in patients treated with immunotherapy than other types of systemic therapy. We, and others, have used bevacizumab to control perilesional edema and worsening radiation necrosis, with variable success, and surgical intervention or laser interstitial thermacoagulation therapy is sometimes needed although caution must be taken with histologies more prone to hemorrhage (54–59). Furthermore, the incidence of seizures from perilesional edema might be decreased with use of prophylactic anti-epileptic medications.

## Future Directions and Conclusion

Use of immune therapy for non-irradiated brain metastases has shown promise in a small number of clinical trials, and requires validation in larger studies and in different tumor types. Experience to date suggests that activity of immune checkpoint inhibitors in brain metastases is similar to that of extracerebral metastases, and exclusion of patients with brain metastases from clinical investigations is no longer justified, although separate studies or separate cohorts for patients with untreated brain metastases might be required. Challenges with treating this patient population include drug-related toxicities such as perilesional edema and tumor-related confounding factors such as necrosis in previously irradiated lesions and intralesional hemorrhage, both of which might require intervention with local or systemic modalities such as surgery, radiation, anticonvulsants, steroids, or VEGF inhibitors. Efficacy of immune checkpoint inhibitors might be further enhanced by combining more than one inhibitor or with combinations with chemotherapy, targeted therapy, or radiation therapy. As the breadth of immunotherapies available for investigation and use expands, predictive biomarkers will also need to be studied and validated. This can be particularly challenging in patients with brain metastases due to the morbidity associated with biopsy; however, if concordance of response is persistently observed as newer drugs are studied in this patient population, extra-cerebral biopsies might suffice. Clinical trials designed specifically for this patient population addressing the effects of multi-modality therapy, particularly combinations of immune checkpoint inhibitors and radiation, are necessary for improving outcomes among individuals with brain metastases.

## Author Contributions

JC and HK wrote this article together.

## Conflict of Interest Statement

The trial of Pembrolizumab in patients with brain metastases from melanoma or non-small cell lung cancer was sponsored by the Yale Cancer Center; partial financial support was provided by Merck, Sharp and Dohme.
